# Multifunctional *π*‐Conjugated Additives for Halide Perovskite

**DOI:** 10.1002/advs.202105307

**Published:** 2022-03-22

**Authors:** Yinan Lao, Shuang Yang, Wenjin Yu, Haoqing Guo, Yu Zou, Zhijian Chen, Lixin Xiao

**Affiliations:** ^1^ State Key Laboratory for Mesoscopic Physics and Department of Physics Peking University Beijing 100871 P. R. China

**Keywords:** additives, halide perovskites, light‐emitting diodes, solar cells, *π*‐conjugation

## Abstract

Additive is a conventional way to enhance halide perovskite active layer performance in multiaspects. Among them, *π*‐conjugated molecules have significantly special influence on halide perovskite due to the superior electrical conductivity, rigidity property, and good planarity of *π*‐electrons. In particular, *π*‐conjugated additives usually have stronger interaction with halide perovskites. Therefore, they help with higher charge mobility and longer device lifetime compared with alkyl‐based molecules. In this review, the detailed effect of conjugated molecules is discussed in the following parts: defect passivation, lattice orientation guidance, crystallization assistance, energy level rearrangement, and stability improvement. Meanwhile, the roles of conjugated ligands played in low‐dimensional perovskite devices are summarized. This review gives an in‐depth discussion about how conjugated molecules interact with halide perovskites, which may help understand the improved performance mechanism of perovskite device with *π*‐conjugated additives. It is expected that *π*‐conjugated organic additives for halide perovskites can provide unprecedented opportunities for the future improvement of perovskite devices.

## Graphical abstract

*π*‐Conjugated additives are competitive for halide perovskite devices. In 3D perovskite, *π*‐electrons with ordered molecular arrangement and distinct interfacial dipole moment can bond with [PbI_6_]^4−^ octahedral to passivate defects and guide crystallization. As for low‐dimensional case, perovskites with *π*‐conjugated ligands exhibit high conductivity and narrow band gap, therefore, charge transfer is greatly promoted.

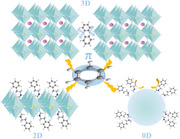

## 1 Introduction

Metal halide perovskites, as promising materials with excellent photoelectrical properties, including excellent light absorption ability,^[^ ^1^ ^]^ high charge carrier mobility,^[^ ^2^ ^]^ long carrier diffusion length,^[^ ^3^ ^]^ adjustable bandgap^[^ ^4^ ^]^ with low trap density and so on, attract high and broad attentions. The organic–inorganic halide perovskite CH_3_NH_3_PbX_3_ (X = Cl, Br, I) were initially researched by Weber in 1987.^[^ ^5^ ^]^ In 1994, perovskite light‐emitting diodes (PeLEDs) appeared when Saito et al. fabricated an organic–inorganic heterostructure electroluminescent (EL) device using a layered perovskite (C_6_H_5_C_2_H_4_)_2_PbI_4_, which can successfully work at liquid‐nitrogen temperature.^[^ ^6^ ^]^ In 1999, perovskite field‐effect transistors with (C_6_H_5_C_2_H_4_)_2_SnI_4_ thin film was first reported by Kagan et al.^[^ ^7^ ^]^ Since the first room temperature PeLED was reported in 2014,^[^ ^8^ ^]^ rapid progress in this field has been witnessed during the past several years. The maximum of external quantum efficiency (EQE) of 3D perovskite‐based PeLEDs was more than 20%.^[^ ^9^ ^]^ Besides, quasi‐2D and quantum dots (QDs)^[^ ^10^ ^]^ perovskites were more appropriate for employing to PeLEDs due to larger exciton binding energy and easier biomolecular recombination.^[^ ^11^ ^]^ Very recently, the maximum EQE was up to 28.0% for green PeLEDs,^[^ ^12^ ^]^ 23.2% for red PeLEDs,^[^ ^13^ ^]^ and 13.8% for blue PeLEDs.^[^ ^14^ ^]^

In the meantime, the study about perovskite solar cells (PSCs) developed rapidly in recent decades since it was first prepared as a sensitizer in 2009 with 3.8% photovoltaic efficiency.^[^ ^15^ ^]^ After then, the first all‐solid‐state PSC was reported in 2012 with perovskite as a light‐absorbing layer, which drew the focus of global attention.^[^ ^16^ ^]^ In 2019, phenethylammonium iodide (PEAI)‐modified planar perovskite solar cell was obtained with a certificated efficiency of 23.3%.^[^ ^17^ ^]^ Recently, the highest reported and certified efficiency of PSCs has reached power conversion efficiency (PCE) as high as 25.8% and 25.7%, respectively..^[^ ^18^ ^]^ By optimizing device structure, choosing appropriate transport layers, adjusting solvent composition and improving film preparation methods, the performance of PSCs has reached rapid progress.^[^ ^19^, ^20^, ^21^, ^22^, ^23^ ^]^

Halide perovskite is a molecular compound of ABX_3_, where A stands for organic cations like CH_3_NH_3_^+^ (MA^+^), NH_2_CH = NH_2_^+^ (FA^+^) or inorganic cations like cesium (Cs^+^), B represents metal cations such as Pb^2+^ or Sn^2+^, while X is halide anions including I^−^, Br^−^, Cl^−^. Except for 3D structures, there are broad explorations in reducing dimensions of perovskite such as 2D/quasi‐2D perovskite, including Ruddlesden–Popper (RP), Doin–Jacobson (DJ) perovskite, and perovskite QDs (0D) by introducing long chain organic molecules. In a word, perovskite can be widely used in PSCs, PeLEDs, photodetectors, etc.

However, multifarious defects still exist in perovskite due to the solution process of perovskite and the subsequent thermal annealing, both on perovskite interface and in interior grain boundaries (GBs), which have negative impacts on device efficiency and stability. On the other hand, to fabricate defect‐free single crystals is still challenging. Therefore, wide range of additives including polymers, small molecules, and ionic compounds are developed as a promising strategy to improve the performance of perovskite devices. As Pb^2+^ can be viewed as Lewis acid and I^−^ as Lewis base, most of the reported additives can be viewed as Lewis acid/base to interact with perovskite via coordination bonds.^[^ ^24^ ^]^ By coordinating with [PbI_6_]^4−^ octahedral, efficient perovskite devices with high quality thin film, negligible hysteresis, and fine long‐term stability have been achieved.

Using additives into perovskite precursor can induce changes in dimension as well. As typical 3D halide perovskite films suffer from severe instability, using low‐dimensional perovskite demonstrates the likelihood of effective problem solving. The introduction of large organic cations provides superior water resistance to protect perovskite. In order to fabricate perovskite devices with excellent performance while reducing dimensions, it is crucial to guarantee well‐aligned organic cations, good film morphology, and most importantly, unhindered carrier transportation.

Up to now, most of the investigated additives are alkyl‐based molecules. One of the major conceptual drawbacks that may be encountered upon introducing an alkyl‐based molecule into the active layer of perovskite involves its potentially insulating nature. Specifically, it would be expected that an alkyl backbone would lack the ability to transfer carriers efficiently through perovskite interface and GBs, and replacing the alkyl group with *π*‐conjugated backbone is a possible solution.

*π*‐Conjugated molecules are a group of molecules containing *π*‐conjugated bonds. As the *π*‐electrons can move freely in the extended molecular orbitals, the *π*‐conjugated molecules are more stable because of lower system energy. Owing to the unique features of coplanar *π*‐electrons and the existence of intermolecular *π*–*π* interaction, the additives usually process superior electrical conductivity, good planarity, and self‐aggregation tendency. Moreover, *π*‐electrons could coordinate with lead atoms in perovskite lattice. Based on the above reasons, *π*‐conjugated molecules have unique influences on perovskite active layers.

The general classification and impact of additives have been reviewed by several groups, and therefore, will not be covered in this article.^[^ ^25^ ^]^ Herein, we first focus on the mechanism and device applications of novel *π*‐conjugated perovskite additives for 3D perovskite in five aspects: defect passivation, lattice orientation guidance, crystallization assistance, energy level rearrangement, and stability improvement. And then, we summarize the main roles of conjugated ligands played in low‐dimensional perovskite devices, both for PSCs and for PeLEDs. Finally, a short perspective on promising future directions of *π*‐conjugated molecules is provided.

## 2 Multifunctional *π*‐Conjugated Additives for 3D Perovskite

**Figure 1:**
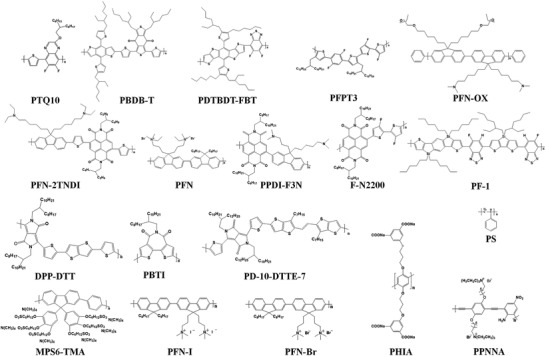
Chemical structure of conjugated polymer additives shown in Table 1.

**Figure 2:**
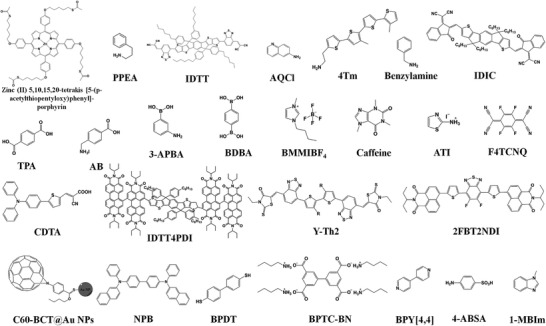
Chemical structure of conjugated additives in Table 2.

*π*‐Conjugated additives are widely employed in 3D perovskite, and remarkable progress has been achieved. In this section, we will summarize the role that conjugated additives play in 3D perovskite by multifunctional mechanisms and how they exert influence on the device performance. The representative additives utilized in PSCs, as one of main applications for perovskite, are listed in **Tables** **1** and **2** and **Figures** **1** and **2**. According to the mechanism of how additives interact with perovskite, we discussed the detailed effect of conjugated molecules in the following parts: defect passivation, lattice orientation guidance, crystallization assistance, energy level rearrangement, and stability improvement.

**Table 1:** Representative polymer additives utilized in 3D PSCs

Additives	Formation process	Perovskite	PCE [%]	*J*_sc_ [mA cm^−2^]	*V*_oc_ [V]	Year
PTQ10	Antisolvent	(FAPbI_3_)_1−_ *_x_* (MAPbI_3_)*_x_*	21.2	23.15	1.12	2018^[^ ^26^ ^]^
PBDB‐T	Antisolvent	CsFAMA‐1^a)^	19.4	22.39	1.11	2018^[^ ^27^ ^]^
PDTBDT‐FBT	Antisolvent	CsFAMA‐2^b)^	18.0	21.62	1.11	2019^[^ ^28^ ^]^
PFPT3	Upper interface	FAPbI_3_	22.0	24.34	1.13	2021^[^ ^29^ ^]^
PFN‐OX	Bottom interface	MAPbI_3_	15.5	20.2	1.02	2015^[^ ^30^ ^]^
PFN‐2TNDI	Upper interface	MAPbI_3−_ *_x_* Cl*_x_*	16.7	21.9	0.98	2016^[^ ^31^ ^]^
PFN	Bottom interface	MAPbI_3_	19.1	21.23	1.11	2017^[^ ^32^ ^]^
PPDI‐F3N	Bottom interface	MAPbI_3_	18.3	22.8	1.09	2017^[^ ^33^ ^]^
F‐N2200	Antisolvent	MAPbI_3_	18.4	22.7	1.06	2018^[^ ^34^ ^]^
PF‐1	Antisolvent	MAPbI_3_	18.7	22.8	1.08	2018^[^ ^34^ ^]^
DPP‐DTT	Antisolvent	CsPbI_2_Br	15.1	15.02	1.29	2019^[^ ^35^ ^]^
PBTI	Antisolvent	CsFAMA‐3^c)^	20.7	22.91	1.13	2019^[^ ^36^ ^]^
PD‐10‐DTTE‐7	Upper interface	MAPbI_3_	18.8	23.30	1.03	2019^[^ ^37^ ^]^
PS	Bottom interface	CsFAMA‐2	17.1	23.55	1.18	2019^[^ ^38^ ^]^
MPS6‐TMA	Bottom interface	CsFAMA‐4^d)^	18.9	21.35	1.12	2020^[^ ^39^ ^]^
PFN‐I	Upper and bottom interface	CsFAMA‐5^e)^	20.5	22.47	1.13	2020^[^ ^40^ ^]^
PFN‐Br	Precursor doping	Cs_0.17_FA_0.87_Pb(I_0.9_Br_0.1_)_3_	20.3	22.92	1.10	2020^[^ ^41^ ^]^
PFN‐Br	Bottom interface	CsFAMA‐5	21.3	21.86	1.18	2020^[^ ^42^ ^]^
PHIA	Precursor doping	MAPbI_3_	20.2	23.92	1.08	2021^[^ ^43^ ^]^
PPNNA	Precursor doping	MAPbI_3_	20.4	23.86	1.12	2021^[^ ^44^ ^]^

^a)^ CsFAMA‐1: Cs_0.04_ FA_0.82_MA_0.14_Pb(I_0.86_Br_0.14_)_3_;

^b)^ CsFAMA‐2: Cs_0.05_ FA_0.80_MA_0.15_Pb(I_0.84_Br_0.16_)_3_;

^c)^ CsFAMA‐3: Cs_0.05_(FA_0.85_MA_0.15_)_0.95_Pb(I_0.85_Br_0.15_)_3_;

^d)^ CsFAMA‐4: Cs_0.05_(FA_0.87_MA_0.13_)_0.95_Pb(I_0.87_Br_0.13_)_3_;

^e)^ CsFAMA‐5: Cs_0.05_ FA_0.79_MA_0.16_Pb(I_0.8_Br_0.2_)_3._

**Table 2:** Representative small molecular additives utilized in 3D PSCs

Additives	Formation process	Perovskite	PCE [%]	*J*_sc_ [mA cm^−2^]	*V*_oc_ [V]	Year
Zinc (II) 5,10,15,20‐tetrakis [5‐(p‐acetylthiopentyloxy)phenyl]‐porphyrin	Upper interface	MAPbI_3_	14.1	21.90	0.93	2016^[^ ^45^ ^]^
PPEA	Upper interface	MAPbI_3_	20.9	23.01	1.15	2019^[^ ^46^ ^]^
IDTT	Antisolvent	CsFAMA‐6^a)^	21.2	23.8	1.19	2020^[^ ^47^ ^]^
AQCl	Precursor doping	CsFAMA‐7^b)^	21.7	22.74	1.18	2021^[^ ^48^ ^]^
4Tm	Upper interface	CsFAMA‐4	22.1	23.79	1.17	2021^[^ ^49^ ^]^
Benzylamine	Upper interface	FAPbI_3_	19.0	20.9	0.91	2016^[^ ^50^ ^]^
IDIC	Upper interface	MAPbI_3_	19.5	22.96	1.11	2017^[^ ^51^ ^]^
TPA	Precursor doping	MAPbI_3_	18.5	23.49	1.05	2017^[^ ^52^ ^]^
AB	Precursor doping	MAPbI_3_ (carbon electrode)	15.6	23.4	0.94	2018^[^ ^53^ ^]^
IDIC	Antisolvent	MAPbI_3_	19.9	22.41	1.12	2018^[^ ^54^ ^]^
3‐APBA	Precursor doping	MAPbI_3−_ *_x_* Cl*_x_*	16.5	23.1	1.1	2019^[^ ^55^ ^]^
BDBA	Precursor doping	MAPbI_3−_ *_x_* Cl*_x_*	17.0	23.4	1.1	2019^[^ ^55^ ^]^
BMIMBF_4_	Precursor doping	CsFAMA‐8^c)^	19.8	23.8	1.08	2019^[^ ^56^ ^]^
Caffeine	Precursor doping	MAPbI_3_	20.3	22.97	1.14	2019^[^ ^57^ ^]^
ATI	Post‐treatment	CsPbI_2_Br	13.9	14.67	1.19	2019^[^ ^58^ ^]^
IDTT4PDI	Post‐treatment	MAPbI_3_	20.1	23.9	1.12	2020^[^ ^59^ ^]^
CDTA	Precursor doping	FASnI_3_	10.2	21.61	0.63	2020^[^ ^60^ ^]^
Y‐Th2	Precursor doping	FA_0.8_MA_0.2_Pb(I_0.8_Br_0.2_)_3_	21.5	23.7	1.14	2020^[^ ^61^ ^]^
Car‐ETTA COF	Bottom interface	CsFAMA‐7	19.8	23.18	1.10	2020^[^ ^62^ ^]^
TFPPy‐ETTA COF	Bottom interface	CsFAMA‐7	19.7	22.50	1.12	2020^[^ ^62^ ^]^
2FBT2NDI	Upper interface	MAPbI_3_	20.1	23.7	1.12	2020^[^ ^63^ ^]^
C_60_‐BCT@Au NPs	Bottom interface	CsFAMA‐7	19.1	22.96	1.14	2020^[^ ^64^ ^]^
NPB	Upper interface	MAPbI_3_	20.2	22.60	1.14	2020^[^ ^65^ ^]^
PDPP‐C20 COF	Upper interface	MAPbI_3_	21.9	24.01	1.13	2020^[^ ^66^ ^]^
BPDT	Precursor doping and upper interface	MAPbI_3_	20.3	22.4	1.15	2021^[^ ^67^ ^]^
BPTC‐BN	Bottom interface	CsFAMA‐9^d)^	21.2	23.90	1.16	2021^[^ ^68^ ^]^
BPY [4,4]	Precursor doping	FA_0.95_MA_0.05_Pb(I_0.9_Br_0.1_)_3_	20.8	24.00	1.05	2021^[^ ^69^ ^]^
4‐ABSA	Precursor doping	CsFAMA‐3	20.3	23.58	1.11	2021^[^ ^70^ ^]^
1‐MBIm	Upper interface	CsFAMA‐3	21.2	23.22	1.15	2021^[^ ^71^ ^]^
F4TCNQ	Precursor doping	Cs_0.09_FA_0.27_MA_0.64_PbI_3_	15.1	22.23	1.07	2021^[^ ^72^ ^]^

^a)^ CsFAMA‐6: Cs_0.05_FA_0.81_MA_0.14_ Pb(I_0.87_Br_0.13_)_3_;

^b)^ CsFAMA‐7: Cs_0.05_(FA_0.88_MA_0.12_)_0.95_Pb(I_0.88_Br_0.12_)_3_;

^c)^ CsFAMA‐8: Cs_0.05_(FA_0.83_MA_0.17_)_0.95_Pb(I_0.9_Br_0.1_)_3_;

^d)^ CsFAMA‐9: Cs_0.05_FA_0.55_MA_0.4_ Pb(I_0.79_Br_0.21_)_3_.

### 2.1 Defect Passivation

It is reported that multifarious defects exist in 3D perovskite during crystal formation,^[^ ^25e^ ^]^ such as organic cation vacancies, halide vacancies, anti‐site substitutions, and impurities. Passivation of such defects through anchoring the appropriate functional groups or molecules to the perovskite lattice or surface could eliminate losses in open‐circuit voltage (*V*_OC_). However, one of the key problems on introducing traditional alkyl‐based passivator into perovskite is its insulating property. Replacing the unconjugated passivator with conjugated ones can solve this problem. As shown in **Table** **3**, the PSCs with conjugated additives exhibit obviously higher short circuit current (*J*_SC_) compared with the unconjugated ones at the same concentrations. The *π*‐conjugated molecules based on the *π*‐conjugated backbone and functional groups could serve as an effective strategy to manipulate trap states and facilitate charge transfer.

Metallic lead is known to be the main nonradiative center and detrimental to the performance of the PSCs. Lots of reported conjugated molecules for PSCs anchor to the perovskite film via incorporation with uncoordinated Pb^2+^ by amino groups,^[^ ^46^, ^49^, ^53^, ^69^ ^]^ carbonyl groups,^[^ ^27^, ^43^, ^51^, ^68^ ^]^ cyano groups,^[^ ^51^ ^]^ sulfur atoms,^[^ ^58^, ^67^ ^]^ or fluorine atoms.^[^ ^28^, ^29^ ^]^ In PeLEDs, poly[(9,9‐bis(3’‐(*N*,*N*‐dimethylamino)propyl)‐2,7‐fluorene)‐alt‐2,7‐(9,9‐dioctylfluorene)] (PFN),^[^ ^73^ ^]^ PFN‐X (I, Br, Cl),^[^ ^74^ ^]^ poly(9,9‐di‐n‐octylfluorenyl‐2,7‐diyl) (PFO),^[^ ^75^ ^]^ poly(9,9‐bis(4′‐sulfonatobutyl)fluorene‐alt‐1,4‐phenylene) potassium (FPS‐K) and poly(9,9‐bis(4′‐sulfonatobutyl)fluorene‐alt‐1,4‐phenylene)tetramethylammonium (FPS‐TMA),^[^ ^76^ ^]^ poly(fluorene‐*co*‐phenylene)‐based anionic conjugated polyelectrolytes,^[^ ^77^ ^]^ and 1,3,5‐*tris*(bromomethyl) benzene (TBB)^[^ ^78^ ^]^ are reported to dramatically improve the device performance by defect passivation. The interaction also exists between bare Pb^2+^ and *π*‐electrons.^[^ ^68^ ^]^ In addition, the additives could form capping layers at the interface when added into antisolvent before thermal annealing. The capping layer prevents organic cations from escaping during thermal annealing and reduced uncoordinated Pb^2+^.^[^ ^26^ ^]^ Additional interaction such as conjugated backbone with surface FA^+^, hydrogen bond between FA^+^/I^−^ and hydroxyl groups are also widely investigated as defect passivation ways.^[^ ^26^, ^52^, ^55^, ^61^, ^67^ ^]^

**Table 3:** Comparison of PSCs with conjugated and unconjugated additives

Perovskite	Unconjugated additives	*J*_sc_ [mA cm^−2^]	Conjugated additives	*J*_sc_ [mA cm^−2^]	Year
AMA_4_PbI_16_	BAI	6.42	AnyI	10.46	2017^[^ ^120a^ ^]^
MAPbI_3_	AVA	22.8	AB	23.4	2018^[^ ^53^ ^]^
MAPbI_3_	PEA	22.03	PPEA	23.01	2019^[^ ^46^ ^]^
MAPbI_3−_ *_x_* Cl*_x_*	4‐ABPA	20.2	BDBA	23.4	2019^[^ ^55^ ^]^
CSFAMA‐9	EDTA‐BN	22.92	BPTC‐BN	23.90	2021^[^ ^68^ ^]^
CsFAMA‐3	1‐MIm	22.91	1MBIm	23.22	2021^[^ ^71^ ^]^
ACs_4_Pb_5_I_16_	EDAI	10.73	PPDAI	14.46	2021^[^ ^121^ ^]^

Similar intermolecular interaction was also found in Sn‐based perovskite. Wu et al. introduced three *π*‐conjugated Lewis base molecules, 2‐cyano‐3‐[5‐(2,4‐difluorophenyl)‐2‐thienyl]‐propenoic acid (CTA‐F), 2‐cyano‐3‐[5‐(2,4‐dimethoxyphenyl)‐2‐thienyl]‐propenoic acid (CTA‐OMe), and 2‐cyano‐3‐[5‐[4‐(diphenylamino)phenyl]‐2‐thienyl]‐propenoic acid (CDTA) to formamidinium tin iodide (FASnI_3_) perovskite.^[^ ^60^ ^]^ It is found that the strong electron‐donating triphenylamine unit on CDTA significantly increases the electron density of Lewis base groups, thus stabilizes the interaction between these groups and Lewis acid SnI_2_ precursor.

It was reported that the molecules with the electron‐donating *π*‐conjugated backbone and electron‐withdrawing functional groups present a quasi‐coplanar configuration and distinct electron delocalization character, attributing to strengthened intermolecular interaction with the positively charged defects in perovskite.^[^ ^46^, ^68^ ^]^ Isikgor et al. demonstrated high binding energy of phenformin hydrochloride (PhenHCl) for the Pb‐deficient surface (−6.78 eV), the I‐deficient (−3.84 eV), and PbI_2_ (−1.67 eV) surfaces by density functional theory (DFT) calculations.^[^ ^79^ ^]^ Because of the electron‐rich and electron‐poor domains in the electron cloud surrounding the molecule, the molecule is more effective for passivation compared with the common additives. What is more, Wang et al. found that benzylamine and phenethylamine (PEA) passivation increases the formation energy of the defects, especially for the surface vacancy, implying the increased energy barrier for the formation of the interface I^−^ vacancies.^[^ ^50^ ^]^ Stronger intermolecular bonding, lowered trap densities, and higher defect formation energy could attribute to denser perovskite film and homogenous surface morphology, resulted in reducing nonradiative recombination and efficient charge transfer, which accounts for higher device efficiency and stability compared with unconjugated additives as reported in lots of works.^[^ ^46^, ^53^, ^55^, ^60^, ^68^, ^70^, ^71^ ^]^

**Figure 3:**
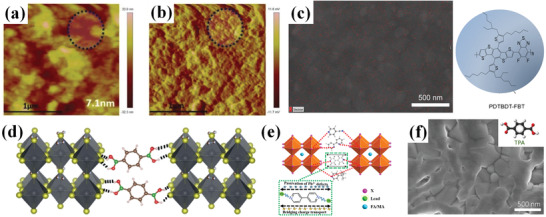
a) Topography and b) amplitude in the EFM images of the perovskite films with CPBDB‐T. Reproduced with permission.^[^ ^27^ ^]^ Copyright 2018, Wiley‐VCH. c) Fluorine EDX mapping of perovskite film prepared by PDTBDT‐FBT treatment (left) and the chemical structure of PDTBDT‐FBT (right). Reproduced with permission.^[^ ^28^ ^]^ Copyright 2019, Wiley‐VCH. d) Scheme of perovskite crystals crosslinked by BDBA with hydrogen bond. Reproduced with permission.^[^ ^55^ ^]^ Copyright 2019, Wiley‐VCH. e) Scheme of Pb^2+^ defect passivation and bridged charge transfer through BPY[4,4]. Reproduced with permission.^[^ ^69^ ^]^ Copyright 2021, American Chemical Society. f) Top view SEM images of MAPbI_3_ films with TPA additive, the insert is the chemical structure of TPA. Reproduced with permission.^[^ ^52^ ^]^ Copyright 2017, American Chemical Society.

Considering the molecule size, there is little chance of incorporation of *π*‐conjugated molecules into perovskite lattice, with the strong interaction of *π*–*π* stacking and high cohesive energy, *π*‐conjugated molecules are highly likely to aligned at GBs. Qin et al. observed molecules gathering areas and reduction of built‐in potential at GBs in amplitude image of electrostatic force microscopy (EFM) (**Figure** **3**a,b) when adding a p‐type *π*‐conjugated polymer poly[(2,6‐(4,8‐bis(5‐(2‐ethylhexyl) thiophen‐2‐yl)‐benzo[1,2‐*b*:4,5‐*b*′] dithiophene))‐alt‐(5,5‐(1′,3′‐di‐2‐thienyl‐5′,7′‐bis(2‐ethylhexyl) benzo[1′,2′‐*c*:4′,5′‐*c′*] dithiophene‐4,8‐dione))] (PBDB‐T) in antisolvent.^[^ ^27^ ^]^ The gathering of additives around perovskite GBs was similarly observed on other conjugated molecules such as fluorinated polymer PDTBDT‐FBT (Figure 3c), poly[(9,9‐bis(3 0‐((*N*,*N*‐dimethyl)‐*N*‐ethyl‐ammonium)‐propyl)‐2,7‐fluorene)‐alt‐2,7‐(9,9‐dioctylfluorene)]di‐iodide (PFN‐I),^[^ ^28^, ^40^ ^]^ poly [(9,9‐bis(3′‐((*N*,*N*‐dimethyl)‐*N*‐ethylammonium)‐propyl)‐2,7‐fluorene)‐*alt*‐2,7‐(9,9‐dioctylfluorene)] dibromide (PFNBr),^[^ ^80^ ^]^ triphenylphosphine oxides (TPPO), etc.^[^ ^81^ ^]^ Moreover, it is found that the PeLEDs employing molecules with more phenyl groups show higher performance. As most defects exist at GBs due to the polycrystalline nature of perovskite film,^[^ ^82^ ^]^ the vacancies are passivated efficiently and the built‐in potential around the charged GBs is weakened.

It was found that some *π*‐conjugated small molecules could permeate through the perovskite layer and assemble with perovskite framework to establish bridge interaction between perovskite grains for faster charge transfer. For example, Nimens et al. investigated the effects of alkyl or *π*‐conjugated boric acid (‐B(OH)_2_) on perovskite (Figure 3d).^[^ ^55^ ^]^ The *π*‐conjugated crosslinking agents were believed to assemble on GBs via strong hydrogen bonding interactions with the exterior iodides of the [PbI_6_]^4−^ octahedral, in this way charges were transferred efficiently through GBs. It was found that the tethering of *π*‐conjugated organic crosslinkers to perovskites yields crystals with prolonged lifetime under continuous light illumination and reduced moisture sensitivity, thus imparting that they are more effective crosslinkers of the perovskite crystals. This bridge interaction was also found by employing a symmetrical conjugated molecular additive (4,4′‐bipyridyl, BPY[4,4]).^[^ ^69^ ^]^ As shown in Figure 3e, BPY[4,4] with molecular length of about 0.7 nm is compatible with perovskite framework (about 0.63 nm). The symmetry structure further facilitates BPY[4,4] molecules assembly on perovskite grains and helped for faster charge transfer. Hou et al. in 2017 incorporated terephthalic acid (TPA) into perovskite precursor solution, which has a rigid phenyl ring and two carboxyl groups.^[^ ^52^ ^]^ The top view scanning electron microscope (SEM) images (Figure 3f) reveal that TPA is easy to gather in the vicinity of perovskite GBs during film formation due to the rigidity property caused by phenyl ring. Thus, some small bar‐shaped or sheet‐shaped grains were formed in the vicinity of GBs and bridged the two sides of bottom perovskite grains. Especially, *π*‐conjugated polymers were reported to infiltrate throughout perovskite films as well even if they are added by post‐treatment. For example, Chen et al. investigated the effect of conjugated polymer additive, poly (bithiophene imide) (PBTI) which is added in antisolvent.^[^ ^36^ ^]^ S element (representing PBTI) was observed in the entire perovskite film from the energy dispersive X‐ray (EDX) mapping, from which the author inferred that the polymer mainly located at GBs. 4‐Aminobenzonitrile (ABN) was also found to penetrate into GBs.^[^ ^83^ ^]^ The *π*‐conjugated molecule‐migration phenomenon can be explained by the strong interaction between [PbX_6_]^4−^ octahedral and the additive.

Another effect *π*‐conjugated molecule had on PSCs is to improve surface contact between perovskite films and charge transport layers (CTLs). Since mesoporous oxide (i.e., mesoporous TiO_2_ and mesoporous ZrO_2_) and hydrophobic hole‐transporting materials (HTMs) are widely used in perovskite devices, the considerable mismatch of surface energy and lattice parameter between hydrophilic perovskite and CTLs occurred in these devices, resulted in poor and random surface coverage. Introducing conjugated additives into perovskite is a solution to alleviate this problem without detrimental effect on charge transfer and device stability. Hu et al. applied an alternative bifunctional conjugated cation 4‐(aminomethyl) benzoic acid hydroiodide (AB) to PSCs. For comparison, the nonconjugated cation, 5‐aminovaleric acid hydroiodide (AVA) and the monofunctional cation, benzylamine (BA) hydroiodide were also investigated in their research.^[^ ^53^ ^]^ BA‐MAPbI_3_ processes faster charge extraction at the perovskite/TiO_2_ interface and faster response from the stabilized power output under maximum power point tracking compared with unconjugated AVA‐MAPbI_3_. In addition, *π*‐conjugated molecules act as interfacial compatibilizer between perovskite and the HTMs are reported to improve the interface wettability and fabricate large and uniform perovskite films.^[^ ^29^, ^32^, ^39^, ^45^ ^]^ It is believed that amphiphilic *π*‐conjugated molecule which has conjugated backbone and hydrophilic functional groups generally matches well with multiple types of CTLs.

In summary, conjugated molecules can coordinate with perovskite via multiple interactions. Due to the influence of *π*‐electrons, conjugated molecules have stronger bonding with perovskite, they can gather at perovskite GBs, even permeate deep into perovskite layer to establish bridge interaction between perovskite grains. Besides, amphiphilic conjugated molecules can improve surface contact between perovskite films and CTLs. Therefore, the introduction of conjugated additives helps with better perovskite defect passivation.

### 2.2 Lattice Orientation Guidance

**Figure 4:**
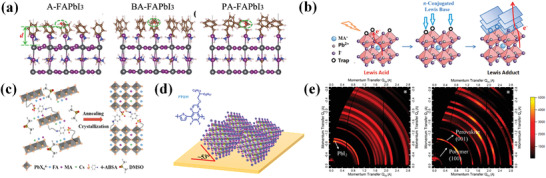
a) DFT simulation of the A‐FAPbI_3_, BA‐FAPbI_3_, and PA‐FAPbI_3_ surface. Reproduced with permission.^[^ ^50^ ^]^ Copyright 2016, Wiley‐VCH. b) Scheme of the interaction of the *π*‐conjugated Lewis base and Pb^2+^. Reproduced with permission.^[^ ^51^ ^]^ Copyright 2017, Wiley‐VCH. c) Scheme of the coordination interaction between 4‐ABSA and perovskite film. Reproduced with permission.^[^ ^70^ ^]^ Copyright 2021, American Chemical Society. d) Schematic diagram of preferential orientation of perovskite crystal after PTQ10 treatment. e) GIWAXS 2D patterns of control FAPbI_3_ (left) and the one treated with PTQ10 during thermal annealing process (right). Reproduced with permission.^[^ ^26^ ^]^ Copyright 2018, American Chemical Society.

There is strong *π*–*π* stacking interaction between *π*‐conjugated molecules in general, considering the high steric hindrance and strong rigidity brought from *π*‐electrons, these additives present coplanarity and highly ordered arrangement with specific orientation when attached to perovskite lattice. Wang et al. showed that the benzyl amine molecules are packed almost perpendicular to the perovskite surface (**Figure** **4**a), while the PEA molecules orient in a relatively irregular fashion.^[^ ^50^ ^]^ By contrast, the indacenodithiophene end‐capped with 1.1‐dicyanomethylene‐3‐indanone (IDIC) thin layers are found to process highly parallel out‐of‐plane *π*–*π* stacking when casted on perovskite (Figure 4b), which is expected to give rise to a small energy disorder of electronic states, and then small *V*_OC_ loss in MAPbI_3_/IDIC‐based PSCs.^[^ ^51^ ^]^

The as‐casted ligand layers localized at the film surface might form interface 2D/quasi 2D structure. The unique electronic property capping layers help to stabilize surface lattice and improve interfacial contact to enhance device efficiencies. For instance, the 2‐(3′′′,4′‐dimethyl‐[2,2′:5′,2″:5″,2′″‐quaterthiophen]‐5‐yl) ethan‐1‐ammonium iodide (4Tm) molecule has been proved to be able to construct into 2D perovskite when mixing together with PbI_2_.^[^ ^84^ ^]^ Ma et al. inserted the interface modifier 4Tm to suppress ion migration and modulate energy level alignment.^[^ ^49^ ^]^ After casting the 4Tm capping layer, grazing‐incidence wide‐angle X‐ray scattering (GIWAXS) pattern shows distinct 2D perovskite formed by replacing organic cations. Dong et al. observed emerging featured peak appeared on the lower angle region when 3‐phenyl‐2‐propen‐1‐amine (PPEA) was designed to post‐treat perovskite photoactive layer, which agrees well with the typical 2D phase (PPEA_2_PbI_4_) in reference.^[^ ^46^ ^]^ Shaper peaks distributed at the azimuth angles between 0° and 180° were observed for PPEA‐treated films in GIWAXS, indicating that relatively ordered orientations of perovskite framework are formed by aniline treatment, where conjugated PPEA cation could encourage more preferable orientation of the bulk perovskite. Recently, an anionic‐conjugated polyelectrolyte containing tetraethylammonium counter ions (MPS2‐TEA)^[^ ^85^ ^]^ was introduced into perovskite precursor and induced a random orientation of quasi‐2D perovskite layers. Their research also shows that there is a large degree of freedom in the additive concentration because of their intrinsic charge transport characteristics.

Because the intermolecular interaction between conjugated additives and the perovskite lattices is extraordinary strong as we discussed above, perovskite preferred orientated growth may be induced by applying *π*‐conjugated additives to perovskite before crystal formation. Cao and co‐workers demonstrated the utilization of *π*‐conjugated 4‐aminobenzenesulfonic acid (4‐ABSA) additive for guiding crystal orientation of perovskite (Figure 4c).^[^ ^70^ ^]^ The 4‐ABSA‐incorporated films exhibit an enhanced diffraction intensity at 14.12°, corresponding to the (110) plane, while the sulfamic acid without a phenyl ring, for comparison, presents no significant enhancement in X‐ray diffraction (XRD) patterns. Therefore, the orientated growth at (110) plane was ascribed to the *π*‐conjugated phenyl ring. Li et al. marked the characteristic peaks at 14.12° with ($\bar{1}11$) plane.^[^ ^28^ ^]^ In their experiments, the perovskite film prepared by dithienobenzodithiophene‐based *π*‐conjugated polymer PDTBDT‐FDT containing antisolvent has more oriented crystals indicated by the larger peak intensity ratio of ($\bar{1}11$) to ($\bar{2}31$). The phenomenon is explained by the authors that the improved grain attachment triggers the faster growing of ($\bar{1}11$)‐oriented grains by consuming neighboring nonoriented crystals. Another conjugated additives poly[(thiophene)‐alt‐(6,7‐difluoro‐2‐(2‐hexyldecyloxy)‐quinoxaline)] (PTQ10) was observed to induce strong azimuthal angle dependency of the (001) peak when coated on this intermediate‐state film (Figure 4d,e).^[^ ^26^ ^]^ Besides, 4,4’‐diaminodiphenyl sulfone, tetrabutylammonium bromide (TBABr), benzyltributylammonium bromide (BTBABr),^[^ ^11^ ^]^ and TPPCl^[^ ^12^ ^]^ were reported to benefit special orientated crystallization of perovskite.

Now that the conjugated molecules have strong interaction with perovskite and present highly ordered orientation as additives, it is probable to serve as template‐agents by forming intermediate adducts with perovskite lattice. Heterogenous nucleation can obviously lower the nucleation free‐energy barrier, thus, the template‐agents control nucleation and crystallization, greatly affect the morphology of perovskite films. As TPA was previously reported as templates for core preorganization,^[^ ^86^ ^]^ Hou et al. incorporated it into perovskite precursor as a supporting template to induce nucleation with lateral growth at GBs.^[^ ^52^ ^]^ Perovskite film with TPA additive without antisolvent treatment shows relatively strong XRD patterns at (110) peak. *π*‐Conjugated molecule ABN,^[^ ^83^ ^]^ PBDB‐T, and polymer with ion side chains (MPS6‐TMA) are used as nucleation site before crystal formation.^[^ ^27^, ^39^ ^]^ Crystal growth is retarded after treatment, GBs turn to be more perpendicular to the substrate, enabling fast hole transfer from the perovskite layer to anode without disturbance.

In conclusion, considering the strong intermolecular interaction with perovskite and the existence of *π*–*π* interaction, conjugated molecules generally present specific orientation on perovskite, which can guide the perovskite lattice orientation during thermal annealing, and some can form interface 2D protecting layers. By guiding the growth of perovskite crystals, crystallization of perovskite is optimized and charge transfer is facilitated.

### 2.3 Crystallization Assistance

**Figure 5:**
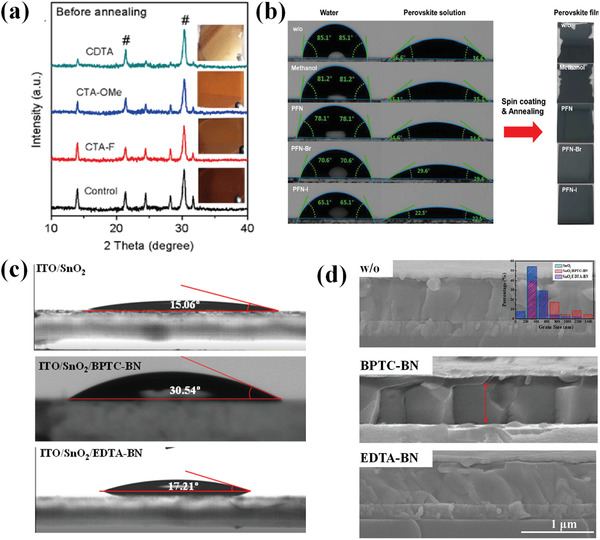
a) XRD patterns of the as‐prepared FASnI_3_ perovskite films treated with different molecules without thermal annealing. The insets showed the photograph of corresponding films. Reproduced with permission.^[^ ^60^ ^]^ Copyright 2020, Springer Nature. b) The contact angle of perovskite solution on ITO glass/poly[*N*,*N*'‐bis(4‐butylphenyl)‐*N*,*N*'‐bis(phenyl)benzidine] with different treatments. From top to bottom are: without treatment (w/o), methanol, PFN, PFN‐Br, and PFN‐I, respectively. Reproduced with permission.^[^ ^40^ ^]^ Copyright 2020, Wiley‐VCH. c) Contact angle between ITO/SnO_2_, SnO_2_/BPTC‐BN, SnO_2_/EDTA‐BN substrates and dimethylformamide (DMF)/dimethyl sulfoxide (DMSO). d) The cross‐section SEM images of perovskite growth on SnO_2_, SnO_2_/BPTC‐BN, and SnO_2_/EDTA‐BN ETLs, inset shows the statistical distribution of grain sizes of devices based on different ETLs. Reproduced with permission.^[^ ^68^ ^]^ Copyright 2021, Elsevier B.V.

The growth mechanism of perovskite crystal can be related to: 1) solution stage for the formation of a solution mixture of a PbI_2_–solvent complex and ABX_3_ intermediates; 2) transition stage from a solution mixture to a solid mixture of intermediates; 3) phase transformation stage from intermediates to a crystalline perovskite.^[^ ^87^ ^]^ The evaporation rate of solvents is critical for the formation of perovskite. Incorporation of *π*‐conjugated additives can dramatically affect the crystallization formation kinetics. The electron‐donating effect of *π*‐conjugated unit causes strong electron delocalization from the conjugated system to the functional groups, which significantly increases the binding strength between additives and perovskite precursors. Stable intermediate adducts might be formed in these cases to slow down crystallization rate of perovskite films. By adding CDTA into FASnI_3_ perovskite before annealing, the film exhibits a nearly amorphous structure with light‐yellow color, indicating the formation of stable intermediate adduct (**Figure** **5**a).^[^ ^60^ ^]^ A low forming energy (*E*_f_) of −104.9 kJ mol^−1^ of the adduct is estimated by DFT calculations, implying that the strong connection between additives and perovskite precursor is beneficial to stabilize the intermediate phase. Intermediate‐state adducts are also founded when adding PTQ10 into perovskite precursor.^[^ ^26^ ^]^ The molecular interactions between polymer and FA^+^ cations retard crystallization. The reduced evaporation rate of solvent molecules will provide a more liquid environment to support the diffusion of ions, allow longer time to form crystal facets with thermodynamically favored orientation, and guarantee the full phase transformation. Taking this advantage, faster charge transport and less nonradiative recombination are expected to occur in the perovskite film, resulting in higher device performance.

Another way that conjugated additives influence perovskite crystallization is by passivating the button interface of perovskite. In the case of perovskite thin film, heterogenous nucleation is the dominant crystallization way. The Gibbs free energy for heterogenous nucleation in perovskite formation process is affected by the contact angle, as expressed by the following equation^[^ ^88^ ^]^

(1) $$\Delta \;G_{\mathrm{heterogenous}}=\;\Delta G_{\mathrm{homogenous}}\times f\left(\theta \right)$$

where *f* (*θ*) = (2 − 3cos*θ* + cos ^3^ *θ*)/4. The *θ* is the contact angle of precursor solution. Therefore, small contact angle will reduce the Gibbs free energy for heterogenous nucleation, thus lowering the nucleation barrier. Strong *π*–*π* stacking interaction may exist between *π*‐conjugated molecules and conjugated backbone of CTLs. Thereby, *π*‐conjugated additives reduce the surface energy by improving surface contact between perovskite film and CTLs. PFN‐I solution is introduced to solve the wettability problem caused by the hydrophobic CTL and facilitate full surface coverage (Figure 5b).^[^ ^40^ ^]^ Larger grain size and higher crystallinity are achieved after PFN‐I treatment, leading to a denser and smoother film morphology. Other conjugated molecules containing hydrophilic conjugated group such as FPS,^[^ ^4^ ^]^ MPS2‐TMA, MPS2‐TEA, MPS2‐TBA^[^ ^85^ ^]^ are deposited at the bottom of perovskite layers with increased surface energy to enhance wettability of perovskite precursor. Conversely, some *π*‐conjugated additives result in nonwetting surface of substrates, leading to decrease of the nucleation sites. Nonwetting surface of biphenyl‐3,3′,5,5′‐tetracarboxylic acid (BPTC, Figure 5c,d) and zinc (II) 5,10,15,20‐tetrakis[5‐(p‐acetylthiopentyloxy) phenyl]‐porphyrin were utilized to passivate defects on the bottom interface and increase nucleus spacing. By suppressing heterogenous nucleation, GB migration in grain growth is promoted and higher peak intensity is observed in XRD, which attributes to increased grain size as well.^[^ ^45^, ^68^ ^]^

In general, conjugated molecules have strong connection with perovskite crystals, they can form intermediates with perovskite and passivate the button interface of perovskite layers. Therefore, the crystallization of perovskite films is much enhanced and device performance is improved.

### 2.4 Energy Level Rearrangement

*π*‐Conjugated additive passivation has significant effects on working function and energy level of the perovskite film, which could be measured by ultraviolet photoelectron spectroscopy (UPS). Specifically, the work functions (*W*_F_) and valence band (VB) level can be determined from the cutoff and Fermi edge of UPS spectra on account of the expression *W*_F_ = 21.22 eV − *E*_cutoff_ and VB = 21.22 eV − (*E*_cutoff_ − *E*_edge_), where *E*_cutoff_ and E_edge_ are the high binding cutoff energy and the near Fermi level edge state energy of the UPS spectra, respectively. In this way, the *W*_F_ and VB for the pristine and modified perovskite films are estimated. It is reported that the change of energy level is a function of the molecular orientation, the ordered conjugated molecules exhibit higher energy level shift compared with the randomly ordered unconjugated molecules.^[^ ^89^ ^]^ Besides, suitable molecular energy level is accessible because multiple molecules with different chemical and electronic structures, including frontier molecular orbital levels and ionic density, are selectable. By narrowing the energy gap between the CTL and perovskite, *π*‐conjugated additives improve the energy‐level alignment on interface.^[^ ^41^ ^]^ Therefore, carrier extraction at the surface interface would be more effective. The well‐matched energy level bending induces a larger built‐in potential, which might concomitantly contribute to increase of *V*_OC_, *J*_sc_, and fill factor (FF).

Specially in PeLEDs, as the deeper highest occupied molecular orbital (HOMO) energy level can match better with the HOMO of blue emitting perovskite layers, PFN,^[^ ^1^ ^]^ PEN‐X (I, Br, Cl),^[^ ^2^ ^]^ FPS‐K, FPS‐TMA,^[^ ^4^ ^]^ and TBB^[^ ^6^ ^]^ were introduced between the HTLs and perovskite layers to improve the interfacial contact and energy alignment. By allowing the effectual hole injection, device performance and stability are improved. Moreover, the electronic structure of injection or transport materials can be modulated by these materials to improve charge injection and balance charge transport, thus avoiding the quenching from interface of perovskite to HTLs.

**Figure 6:**
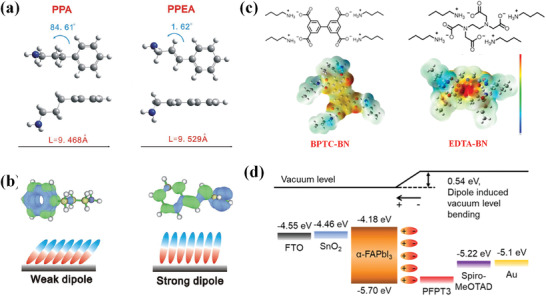
a) Geometric optimizations of PPA (left) and PPEA (right). b) ESP of PPA (left) and PPEA (right) calculated by DFT (electronegative part is green and electropositive part is blue). Reproduced with permission.^[^ ^46^ ^]^ Copyright 2019, Wiley‐VCH c) The electron density of BPTC‐BN and EDTA‐BN calculated by DFT. Reproduced with permission.^[^ ^68^ ^]^ Copyright 2021, Elsevier B.V. d) Experimentally determined energy‐level diagrams of the interface between the perovskite and PFPT3. Reproduced with permission.^[^ ^29^ ^]^ Copyright 2021, American Chemical Society.

It is worth noticing that stronger interfacial dipole moment can be induced by conjugated molecules compared with unconjugated ones. Most conjugated backbones are electron‐donating while the functional groups are electron‐withdrawing, thus, the additives present electron delocalization. The favorable molecule orientation (as we discussed above), intense intermolecular interaction and distinct electron delocalization of conjugated additives contribute to stronger dipole moment. Garai and co‐works reported strong local electron density around the side‐chain carboxylate ions on poly(p‐phenylene)‐based molecule PHIA from the electrostatic potential (ESP) profile.^[^ ^43^ ^]^ Zhang et al. observed quadrupole moments on conjugated molecule BPTC and similar but unconjugated molecule ethylene diamine tetraacetic acid (EDTA) (**Figure** **6**c).^[^ ^68^ ^]^ The conjugative effect between carboxylic ion and *π*‐electron form high electron cloud density of carboxylic ion on BPTC, resulting in higher charge‐quadrupole interaction energy. Dong and co‐workers explored the origin of the interfacial dipole by calculating geometric optimizations of conjugated molecule PPEA and less‐conjugated molecule 3‐phenylpropylamine (Figure 6a,b) by DFT.^[^ ^46^ ^]^ As the PPEA molecule shows a quasi‐coplanar structure with the smaller tilted angle, while the less‐conjugated 3‐phenylpropylamine (PPA) exhibits a relative distorted configuration, ESP shows that obvious electronic delocalization occurred in PPEA inducing a larger component of dipole moment normal to the surface (2.82 D), whereas PPA emerges in an entangled state. Moreover, the existence of interfacial dipole moment is manifested by Lyu et al. when investigating the fluorinated conjugated polymer 5,5′‐(2,5‐difluoro‐1,4‐phenylene)bis(3‐[2‐decyltetradecyl]‐thiophene) and 3,3′‐difluoro‐2,2′‐bithiophene (PFPT3) as an interfacial layer between perovskite and HTL.^[^ ^29^ ^]^ It is revealed that the electron density of the Pb in the perovskite structure decreased upon formation of the junction with PFPT3 and that the electron density of F in the polymer simultaneously increased because of electron transfer from the perovskite layer to PFPT3. As a result, the deposition of the perovskite using a low‐concentration PFPT3 (0.01 mg mL^−1^) led to a remarkable increase in the vacuum level (e.g., *ϕ* = 0.54 eV) (Figure 6d). Furthermore, PFO^[^ ^3^ ^]^ was reported to act as a donor to irradiate with the light energy then induce the oscillating dipole of perovskite. As a result, the nonradiative energy transfer is promoted. The strong interfacial dipole moment induced by *π*‐electrons further improves the band alignment and reinforces the built‐in electric force across the interface, thereby facilitating the charge transfer and providing obvious improvements in *V*_OC_.

To sum up, the introduction of conjugated molecules helps with better energy level alignment and stronger interfacial dipole moment. By optimizing the energy arrangement of perovskite devices, charge transport has been much more balanced.

### 2.5 Stability Improvement

Perovskite devices with conjugated additives have been reported to improve the moisture, thermal, and light stability. The mechanism of the improved stability can be summarized as follows.

First, moisture penetration is prevented due to the improved device hydrophobicity. In the case of MAPbI_3_, the degradation reaction proceeds first by generating perovskite hydrate. This hydrated perovskite phase then degrades to MAI and PbI_2_. When MAPbI_3_ is exposed to UV light in the presence of oxygen and moisture, degradation is found to accelerate compared with degradation in dark conditions.^[^ ^90^ ^]^ The hydrophobic conjugated backbones and functional groups provide an additional level of barrier against ambient moisture.^[^ ^40^ ^]^ Perovskite crystal with a stronger bonding interaction with *π*‐conjugated additives will be more resistant to degradation because they are kinetically less susceptible to water. In certain situations, interfacial 2D layers are formed, further offering enhanced protection to perovskite films.

Second, with grain boundary passivation of *π*‐conjugated molecules, the kinetic barrier of perovskite degradation is improved, which prevents phase transition, reduces ion migration, and improves the device stability. Considering the first‐order kinetics for the decomposition of perovskite compounds, and using the integrated form of the rate equation^[^ ^91^ ^]^

(2) dn/dt=−kn

i.e.

(3) $$k\;=\;\ln\left(\frac{n_{1}}{n_{2}}\right)/\left(t_{2}-t_{1}\right)$$

where *k* is the kinetic constant, and *n*_1_ and *n*_2_ are the number of moles of reactant at times *t*_1_ and *t*_2_, respectively. The conjugated molecules lock with perovskite crystals, especially on GBs, thus, kinetic constant is lowered and ion migration is hindered. In this way, the decay process is greatly suppressed with higher thermal resistance and less *J*–*V* hysteresis. Additive‐based devices such as poly(bithiophene imide) (PTBI), PBDB‐T, and IDIC with GBs coverage are found to be much more stable w/o light illusion.^[^ ^27^, ^36^, ^51^ ^]^ Wang et al. observed the evident of suppressed ion migration by EDX mapping of PSC devices after caffeine addition.^[^ ^57^ ^]^ Caffeine molecule was reported as “molecule lock” to bond with perovskite crystals utilizing carboxyl groups in different chemistry environment, compared with bare perovskite powder, the sublimation temperature of MAI and PbI_2_ increases, implying the high binding energy between caffeine and the precursor.

In addition, when added before thermal annealing, *π*‐conjugated additives such as PTQ10 preserve the escape of organic cations and help fabricate perovskite films with a balanced stoichiometric phase, further preserve the decomposition in thermal test.^[^ ^26^ ^]^ Moreover, researchers found that the perovskite/conjugated PFPT3/doped 2,2',7,7'‐tetrakis[*N*,*N*‐di(4‐methoxyphenyl)amino]‐9,9'‐spirobifluorene (Spiro‐OMeTAD) samples maintained its dark color longer than perovskite/Spiro‐OMeTAD with hydrophobic additives ones. This experiment suggests that the adhesion on perovskite interface plays a more important role in device stability than the CTL's hydrophobic. The increased adhesive interfacial contact between the CTLs and perovskite layers generated from amphipathic conjugated molecules greatly contributes to device stability.

By increasing the moisture resistance of perovskite films, suppressing the perovskite degradation on GBs and acting as a capping layer on perovskite to prevent the escape of organic cations, conjugated additives improve the moisture, thermal, and light stability of perovskite devices. What is more, due to the good conductivity of conjugated molecules, interface layers which are thick enough to protect perovskite layers can be formed without hindering charge transport.

## 3 Multifunctional *π*‐Conjugated Additive/Ligand for Low‐Dimensional Perovskite

**Figure 7:**
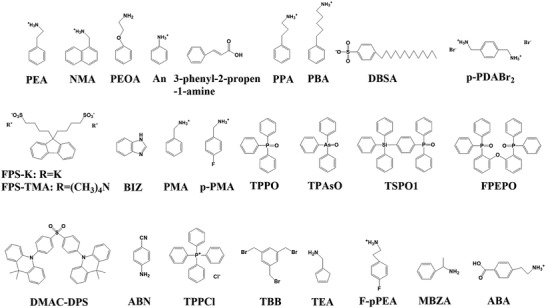
Chemical structure of conjugated additives shown in Table 4.

The instability of 3D perovskite against moisture, light, and heat is a troubling challenge in practical application. In response, low‐dimensional organic–inorganic halide perovskite has emerged as a promising alternative approach to enhance the perovskite stability over the past decades. Moreover, as the density of the electronic states can be widely tuned by adjusting the crystal shape, composition, and size, significantly different properties can be achieved for low‐dimensional perovskite ligands. There have been lots of reports about low‐dimensional perovskite that incorporates the aliphatic ammonium cations, however, only a few researches regarding conjugated organic cations. Unlike the optically transparent and electrically insulting property of traditional alky‐based organic ligands, molecules with *π*‐electrons can offer unique characters such as electronic conductivity and light absorption, therefore, low‐dimensional perovskite with tailored *π*‐conjugated ligands has various applications in PSCs, PeLEDs, photodetectors, and so on. The representative additives reported in low‐dimensional PeLEDs are listed below in **Table** **4** and **Figure** **7**. In this section, we discussed the characters of *π*‐conjugated molecules on low‐dimension perovskites from three aspects: enhancing out‐of‐plane conductivity, modulating energy alignment, and improving structural stability.

**Table 4:** Representative additives utilized in low‐dimensional PeLEDs

Additives	Formation process	Perovskite	Dimension	Peak wavelength [nm]	EQE	Current efficiency [cd A^−1^]	Year
PEAI	Precursor doping	MAIPbI_3_	Quasi‐2D	/	8.8%	/	2016^[^ ^92^ ^]^
NMAI	Precursor doping	FAPb(I/Br)_3_	Quasi‐2D	/	11.8%	/	2016^[^ ^93^ ^]^
POEA	Precursor	MAPbBr_3_	Quasi‐2D	524	2.82%	8.23	2017^[^ ^94^ ^]^
An‐HI	Ligand exchange	CsPb(Br/I)_3_	0D	645	14.1%	11.6	2018^[^ ^95^ ^]^
PFN	Bottom interface	CsPbBr_3_	Quasi‐2D	512	14.4%	45.2	2018^[^ ^73a^ ^]^
PEAI	Precursor doping	CsPbI_3_	0D	516	14.1%	14.5	2018^[^ ^96^ ^]^
3‐Phenyl‐2‐propen‐1‐amine	Ligand exchange	MAPbBr_3_	0D	517	/	9.08	2018^[^ ^97^ ^]^
3‐phenyl‐2‐propen‐1‐amine	Ligand exchange	CsPbBr_3_	0D	/	7.13%	23.5	2018^[^ ^98^ ^]^
PEABr	Ligand exchange	CsPbBr_3_	0D	518	1.79%	5.91	2018^[^ ^99^ ^]^
PPABr	Ligand exchange	CsPbBr_3_	0D	516	2.47%	7.93	2018^[^ ^99^ ^]^
PBABr	Ligand exchange	CsPbBr_3_	0D	514	4.33%	13.4	2018^[^ ^99^ ^]^
PBABr	Precursor doping	Cs*_x_* FA_1−_ *_x_* PbBr_3_	Quasi‐2D	483	9.50%	12.0	2019^[^ ^100^ ^]^
DBSA	Precursor doping	CsPbBr_3_	0D	515	/	/	2019^[^ ^101^ ^]^
P‐PDABr_2_	Precursor doping	P‐PDAPbBr_4_	2D	465	2.60%	/	2019^[^ ^102^ ^]^
P‐PDABr_2_	Precursor doping	FA*_n_*_−1_Pb*_n_* X_3_ *_n_*_+1_	Quasi‐2D	776	5.20%	/	2019^[^ ^103^ ^]^
F‐PMA	Precursor doping	MAPb_0.4_Sn_0.6_I_3_	Quasi‐2D	917	5.00%	/	2019^[^ ^104^ ^]^
FPS‐K	Bottom interface	PMA_2_FA_2_Pb_3_Br_10_	Quasi‐2D	540	6.50%	27.6	2019^[^ ^76^ ^]^
FPS‐TMA	Bottom interface	PMA_2_FA_2_Pb_3_Br_10_	Quasi‐2D	542	10.2%	43.6	2019^[^ ^76^ ^]^
BIZ	Precursor doping	FAPbBr_3_	Quasi‐2D	530	7.70%	/	2019^[^ ^105^ ^]^
PMA	Precursor doping	MAPbBr_3_	3D/2D hybrid	/	4.23%	20.6	2020^[^ ^106^ ^]^
TPPO	Antisolvent	PEA_2_Cs_2.4_MA_0.6_Pb_4_Br_13_	Quasi‐2D	517	14.0%	/	2020^[^ ^107^ ^]^
TPAsO	Antisolvent	PEA_2_Cs_2.4_MA_0.6_Pb_4_Br_13_	Quasi‐2D	517	8.80%	/	2020^[^ ^107^ ^]^
PFN‐X(I/Br/Cl)	Bottom interface	CsPbBr*_x_* Cl_3−_ *_x_*	0D	470	1.34%	1.24	2020^[^ ^74^ ^]^
MPS2‐TMA	Bottom interface	PEA_2_FA_2_Pb_3_Br_7_Cl_3_	Quasi‐2D	489	1.89%	3.02	2020^[^ ^77^ ^]^
MPS2‐TEA	Bottom interface	PEA_2_FA_2_Pb_3_Br_7_Cl_3_	Quasi‐2D	489	2.04%	3.13	2020^[^ ^77^ ^]^
MPS2‐TBA	Bottom interface	PEA_2_FA_2_Pb_3_Br_7_Cl_3_	Quasi‐2D	489	2.60%	4.21	2020^[^ ^77^ ^]^
TSPO1	Bilateral interface	CsPbBr_3_	0D	516	18.7%	75.0	2020^[^ ^108^ ^]^
DPEPO	Bilateral interface	CsPbBr_3_	0D	516	17.1%	66.0	2020^[^ ^108^ ^]^
TPPO	Bilateral interface	CsPbBr_3_	0D	516	17.8%	71.0	2020^[^ ^108^ ^]^
DMAC‐DPS	Bilateral interface	CsPbBr_3_	0D	516	12.8%	63.0	2020^[^ ^108^ ^]^
ABN	Ligand exchange	CsPbBr_3_	0D	534	8.85%	26.6	2020^[^ ^83^ ^]^
TPPCl	Antisolvent	PEA_2_Cs_5_Pb_5_Br_17_	Quasi‐2D	520	19.1%	/	2020^[^ ^109^ ^]^
TBB	Bottom interface	FAPbBr_3_	0D	531	20.1%	77.2	2020^[^ ^110^ ^]^
TEAI	Precursor doping	TEA_2_SnI_4_	2D	638	0.62%	/	2020^[^ ^111^ ^]^
PEABr	Precursor doping	FAPbBr_3_	Quasi‐2D	548	12.4%	52.1	2020^[^ ^112^ ^]^
NMABr	Precursor doping	FAPbBr_3_	Quasi‐2D	548	3.4%	16.3	2020^[^ ^112^ ^]^
PMABr	Precursor doping	(CsBr)_0.9_(MABr)_0.1_PbBr	Quasi‐2D	/	4.91%	/	2021^[^ ^113^ ^]^
PEABr	Precursor doping	(CsBr)_0.9_(MABr)_0.1_PbBr	Quasi‐2D	/	9.35%	/	2021^[^ ^113^ ^]^
PBABr	Precursor doping	(CsBr)_0.9_(MABr)_0.1_PbBr	Quasi‐2D	/	4.35%	/	2021^[^ ^113^ ^]^
F‐pPEABr	Precursor doping	MAPbBr_3_	Quasi‐2D	520	20.4%	/	2021^[^ ^114^ ^]^
PPAI	Precursor doping	FA_0.83_Cs_0.17_PbI_3_	Quasi‐2D	789	17.5%	/	2021^[^ ^115^ ^]^
ABA	Precursor doping	PEA*_x_* PA2_−_ *_x_* (CsPbBr_3_)*_n_*_−1_PbBr_4_	Quasi‐2D	486	10.1%	11.9	2021^[^ ^116^ ^]^
PEA	Precursor doping	CsPbBr_3_	0D	470	4.70%	/	2021^[^ ^117^ ^]^
PFNBr	Precursor doping	PEA_2_PbCl*_x_* Br_(4−_ *_x_*_)_ *x*Br	Quasi‐2D	485/476	11.2/8.0%	/	2021^[^ ^118^ ^]^
MBZA	Precursor doping	(POEA*_x_* MBZA_1−_ *_X_*)Cs*_n_*_−1_PbI_3_ *_n_*_+1_	Quasi‐2D	653	11.5%	5.7	2021^[^ ^119^ ^]^

### 3.1 Enhancing Out‐of‐Plane Conductivity

**Figure 8:**
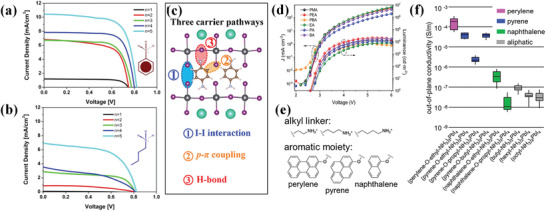
*J*−*V* curves of the champion device prepared with a) AnyIPb*_n_* and b) BAIPb*_n_* 2D/3D layers. Reproduced with permission.^[^ ^120a^ ^]^ Copyright 2017, American Chemical Society. c) Illustration of the three carrier transport channels between PPDACs*_n_*_−1_PbnI_3_ *_n_*_+1_ interlayer. Reproduced with permission.^[^ ^121^ ^]^ Copyright 2021, Wiley‐VCH. d) *J*–*V*–*L* curves for the control and treated devices. Reproduced with permission.^[^ ^122^ ^a]^ Copyright 2018, Springer Nature. e) Modular molecular design of the ammonium iodide cation. f) Conductivity of nine different layered perovskites with either an aliphatic cation or a cation containing naphthalene, pyrene, or perylene. Reproduced with permission.^[^ ^126^ ^]^ Copyright 2018, American Chemical Society.

The out‐of‐plane conductivity is the conductivity in the out‐of‐plane direction. The depressed current density and device performance in low‐dimensional PSCs are mainly owing to the inhibition of out‐of‐plane charge transfer. Based on *π*‐conjugated aromatic spacers, the out‐of‐plane charge transfer of 2D perovskite is greatly enhanced. Rodríguez‐Romero et al. compared the photovoltaic behavior of 2D/3D perovskite (C_6_H_5_NH_3_)_2_(MAI)*_n_*_−1_(PbI_2_)*_n_* (denoted as AnyIPb*_n_*) with typically layered perovskite (BANH_3_)_2_(MAI)*_n_*_−1_(PbI_2_)*_n_* (denoted as BAIPb*_n_*).^[^ ^120^ ^]^ By the incorporation of a bulky cation with unlocalized and polarizable *π*‐electrons, the dielectric contrast between organic and inorganic layers is modulated with reduced exciton binding energy. Benefiting from the lowered Coulomb interaction between carriers and improved charge transport, significant improvement was observed at the photocurrent and electroluminescence (**Figure** **8**a,b). Fang et al. reported a quasi‐2D DJ perovskite via a *π*‐conjugated aromatic spacer p‐phenylenediamine (PPDA).^[^ ^121^ ^]^ Compared with the alkyl diamine spacer ethylenediamine (EDA), the mechanism for improving out‐of‐plane charge transport of (PPDA)Cs*_n_*_−1_Pb*_n_* I_3_ *_n_*_+1_ is elucidated as follows. First, attributing to the existence of high charge density around terminal I ions, I–I interaction between the adjacent inorganic layers is quite strong. Second, as the direction of phenyl ring of PPDA is nearly paralleled with the p*_x_* and p*_y_* orbitals of I ions, there are p‐*π* electronic coupling between PPDA and the terminal I ions. Finally, electron redistribution suggests the strong hydrogen bonds between PPDA and the bridge I ions. These three effects induce new carrier channels for fast out‐of‐plane charge transport (Figure 8c).

Similarly, the incorporation of *π*‐conjugated molecules also improves the carrier mobility of PeLEDs. Conjugated ligands with one phenyl ring, e.g., phenylmethylamine (PMA), propylamine (PA), PEA, and phenylbutylamine, were reported to exhibit higher current density in quasi‐2D perovskite compared with unconjugated ligands of ethylamine (EA) and BA (Figure 8d).^[^ ^122^ ^]^ Recently, PFN‐Br is reported as the ligand of blue‐emitting perovskite to benefit the hole injection and carrier transport from the GBs to the inorganic layer [PbBr_6_]^4−^ of quasi‐2D perovskite.^[^ ^80^ ^]^ In perovskite QDs, to replace the long alkyl chain ligands of oleylamine and oleic acid, conjugated molecules such as PEA,^[^ ^123^ ^]^ 3‐phenyl‐2‐propen‐1‐amine,^[^ ^98^ ^]^ 3,3‐diphenylpropylamine (DPPA),^[^ ^124^ ^]^ and dodecylbenzene sulfonic acid (DBSA)^[^ ^101^ ^]^ are employed to bond with the residual uncoordinated sites and facilitate in situ exchange on the QDs surface. Moreover, the conjugation of ligands can be enhanced to help with the formation of more effective charge carrier transport channels by the incorporation of electron‐withdrawing (‐COOH, ‐F) and electron‐donating groups (‐NH_2_). Para‐position substitution of phenyl ring ligands including 1,4‐bis(aminomethyl) benzene,^[^ ^102^, ^103^ ^]^ p‐fluorophenethylammonium (F‐pPEA),^[^ ^125^ ^]^ and 4‐(2‐aminoethyl) benzoic acid (ABA) are introduced into perovskite to obtain PeLEDs with enhanced radiative recombination.

On the other hand, the energy level and transport behavior of carriers of perovskite active layers can be precisely tuned by systematically modulating the conjugated spacer. A set of bis(aminoethyl)‐quaterthiophene‐based lead bromide AEnTPbBr_4_ was studied by Liu et al. to explore the ability to tune quantum‐well nature by varying the number *n* of organic components.^[^ ^127^ ^]^ It turned out that the organic lowest unoccupied molecular orbital (LUMO) decreases as *n* increases, while the inorganic LUMO increases with *n*. The results further demonstrate that when adding more conjugated groups to the system, the optoelectronic coupling between organic and inorganic systems will be enhanced. The predicted AEnTPbBr_4_ compounds for *n* = 2–5 exhibit type‐IIb level alignments while the *n* = 1 compound yields a type‐Ib alignment. A study carried out by Passarelli et al. reveals the formation of conjugated compounds has great influence on conductivity (Figure 8e).^[^ ^126^ ^]^ By performing conductivity measurement on multiple layered perovskite crystals, they found the conductivity spans about four orders of magnitude (Figure 8f). Layered perovskite with aliphatic cations unsurprisingly has the lowest conductivity, and naphthalene‐containing material follows closely. Pyrene and perylene‐based materials were found to have the highest conductivity. The authors contribute the difference in conductivity to whether the organic spacers have proper energy alignment with perovskite lattice.

In Passarelli's work mentioned above, the conductivity difference between different alkyl linker of the organic cations is glaringly obvious as well (Figure 8f).^[^ ^126^ ^]^ Although not as dominant as the conjugated components, there is distinct conductivity difference between them. Generally, on the same order, the out‐of‐plane conductivity slightly decreases when increasing the layer spacing. Different trend observed in pyrene‐containing crystals was attributed to structure formation changes. The low‐conductive pyrene‐containing materials with propyl group exhibit an edge‐to‐edge arrangement for organic cations in crystal structure while other materials exhibit an edge‐to‐face type *π*‐interaction.

To sum up, the out‐of‐plane conductivity of low‐dimensional perovskite can be enhanced greatly with conjugated ligands. It is reported that conductivity mostly depends on the conjugated backbones, which is determined by the alignment of organic cations and the number of conjugated units. Not as dominant as the conjugated components, different alkyl linkers also lead to a difference in conductivity.

### 3.2 Modulating Energy Alignment and Electronic Coupling

**Figure 9:**
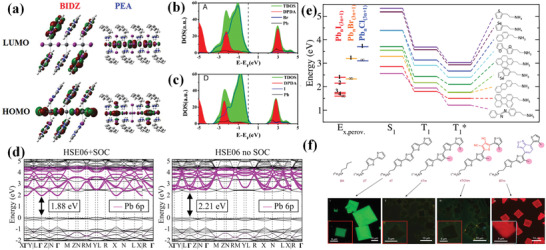
a) DFT‐calculated LUMO and HOMO of BIDZPbI_4_ and (PEA)_2_PbI_4_. Reproduced with permission.^[^ ^128^ ^]^ Copyright 2021, Wiley‐VCH. Projected partial density of state (PDOS) of b) (DPDA)PbBr_4_ and c) (DPDA)PbI_4_. Reproduced with permission.^[^ ^129^ ^]^ Copyright 2017, American Chemical Society. d) The electronic band structures of AE4TPbBr_4_, calculated by DFT‐HSE06+SOC and DFT‐HSE06, respectively. Reproduced with permission.^[^ ^127^ ^]^ Copyright 2018, American Physical Society. e) Alignment between perovskite exciton levels, organic T_1_ excitation energies, and organic T_1_^*^ excited‐state geometry emission energies for a select subset of examined organic spacers. Reproduced with permission.^[^ ^131^ ^]^ Copyright 2019, American Chemical Society. f) The chemical structures of the conjugated organic ligands BA, 2T, 4Tm, 4TCNm, BTm and the corresponding PL images of their assembled 2D crystals under ultraviolet excitation. The insets are the PL images for the corresponding monolayer‐thick single‐quantum‐well structures. Reproduced with permission.^[^ ^84^ ^]^ Copyright 2019, Springer Nature.

In lead halide perovskite, the HOMO is mainly formed by halide orbits, while the LUMO is made up from the composition of lead/halide. In layered perovskites with conjugated organic spacers, it is claimed that the *π*‐electrons have significant contribution to the lowered LUMO by DFT calculation. Fu et al. compared the energy level structure of quasi‐2D perovskite with fully conjugated aromatic 2,2‐biimidazolium (BIDZ) cation and well‐known organic cation PEA.^[^ ^128^ ^]^ The LUMO of BIDZPbI_4_ mainly distributes at BIDZ, whereas the LUMO of (PEA)_2_PbI_4_ distributes at perovskite slabs (**Figure** **9**a). The incorporation of conjugated organic cations greatly enhanced the *J*_SC_ from 10.4 mA cm^−2^ (PEA‐based) to 16.1 mA cm^−2^ (BIDZ‐based). Hautzinger et al. performed investigation into the contribution of *N*,*N*‐dimethylphenylene‐*p*‐diammonium (DPDA) dications orbits on (DPDA)PbBr_4_ and (DPDA)PbI_4_.^[^ ^129^ ^]^ The DPDA dications similarly have great contribution to the LUMO (Figure 9b,c). This result differed from most of the reported 2D perovskite materials that both the HOMO and LUMO localize on the inorganic layers. The band gap of layered perovskite is lowered and the electronic communication between the organic and inorganic layers is promoted, resulting in higher carrier mobility and more favorable photovoltaic performance.

However, the DFT calculations mentioned above do not include spin–orbit coupling (SOC) correction. Other studies indicated that when SOC correction is included, the results about what extent *π*‐conjugated organic layers participate in the energetic ordering of states near the band extrema will be totally different. Leveillee et al. predicted the electronic structure of low‐dimensional perovskites with two single‐ring organic spacers, ammonium‐propyl‐imidazole (API) and PEA.^[^ ^130^ ^]^ Once SOC is neglected, the LUMO would be attributed to mostly API *π* ^∗^ states as a band inversion occurs at the Г point. In contrast, if SOC is taken into account, the band ordering at the Г‐point LUMO changes without band inversion but the API *π* ^∗^ states still lowered the Pb:p state energy. Liu et al. also noticed the significance of SOC in first‐principle DFT calculation when 2D perovskite is with conjugated organic spacer, bis(aminoethyl)‐quaterthiophene lead bromide AE4TPbBr_4._^[^ ^127^ ^]^ The nature of the LUMO changed from organic to inorganic and the band gap reduces about 0.3 eV when SOC correction is taken into calculation (Figure 9d). Although the role conjugated cations played in the LUMO is still controversial, there is no doubt that compared with unconjugated organic ones, conjugated cations have much greater contribution to the conduction band. The *π* and *π* ^*^ states located in the near‐edge conduction band facilitate electronic coupling between organic and inorganic layers.

It is crucial to choose conjugated ligands with suitable energy states for the design of efficient PeLEDs. Resulted from the high dielectric constant and strong SOC, the delocalization of first singlet (S_1_) and triplet (T_1_) Wannier–Mott excitons are hardly distinguishable in optical emission spectra within inorganic perovskite layers. Instead, T_1_ excitation energy is distinctly lower than S_1_ excitation energy in organic layers. If the S_1_ of conjugated ligand is lower than that of inorganic layer, efficient triplet–triplet Dexter energy transfer happens from inorganic layers to the triplet states of conjugated ligands, which leads to nonradiative recombination. It is found that the number of phenyl rings has significant influence on the triplet energy level. In general, conjugated ligands with one phenyl ring are expected to have higher triplet energy level than the ones with more phenyl rings. For example, the triplet energy levels of benzene and naphthalene are estimated to be 3.8 and 2.6 eV, respectively.^[^ ^132^ ^]^ As a result, naphthalene‐based ligand 1‐naphthylmethylamine (NMA) shows lower triplet level (2.6 eV) than the benzene‐based ligand PEA (3.6 eV).^[^ ^133^ ^]^ Besides, as thiophene exhibits a high triplet level (3.75 eV), thiophene‐based ligands 2‐thiophene ethyl ammonium (TEA^+^) and 2‐thiopene methyl ammonium (TMA^+^) are employed to fabricate PeLEDs with green emission.^[^ ^134^ ^]^ What is more, the derivatives of oxadiazole^[^ ^135^ ^]^/azole‐based^[^ ^136^ ^]^ material such as quinolone^[^ ^137^ ^]^ show higher triplet energy level than the ones with one phenyl ring.^[^ ^138^ ^]^ If these ligands with appropriate alkyl chains are introduced into perovskite, the performance of PeLEDs may be further improved. By tailoring the conjugated backbone, the triplet energy level can be raised to effectively block the triplet excitons from perovskite.

As for PSCs, the HOMO–LUMO gap of organic layers can be further lowered by adding more conjugated groups to the system. Thereby, the energy level alignment can change from type‐I to type‐II, promoting carrier extraction from perovskite layers to organic layers. One way to demonstrate energy transfer is to observe triplet emission from organic spacers. Charge transfer may happen when T_1_ in organic layer approaches the energy of bound exciton in perovskite layer. A study carried out by Leveillee et al. compared the S_1_ and T_1_ of a set of 2D perovskite materials with different conjugated organic cations by time‐resolving DFT calculation.^[^ ^131^ ^]^ In general, as shown in Figure 9e, the energy level of S_1_ and T_1_ are lowered when adding the number of conjugated groups. Gao et al. observed the energy transfer from perovskite layer to organic layer.^[^ ^84^ ^]^ 2D perovskite with conjugated ligands, 4Tm and 4TCNm (Figure 9f), exhibited quenched photoluminescence (PL) under UV irradiation, whereas (2T)_2_PbI_4_ showed a green color and (BTm)_2_PbI_4_ showed a red color. The green and red colors are originated from the inorganic and organic layers, respectively. In contrast, the PL results of (4Tm)_2_PbI_4_ and (4TCNm)_2_PbI_4_ indicate their type‐II energy alignment, which is consistent with the following calculation. The promoted charge separation at interface mentioned above is beneficial to photovoltaic applications and triplet emission.

Similarly, conjugated molecules are applied to promote charge separation in QD PSCs as well. In conventional QD PSCs, the exciton binding energy is much higher than that of 3D perovskite. As a result, the carriers are likely to recombine before they reach the extraction layers. *π*‐Conjugated molecules with satisfying conductivity are employed into perovskite QDs to form hetero‐interface to enhance carrier extraction. In the meanwhile, the tradeoff between perovskite QD stability and carrier transport is well addressed. As conjugated organic spacers are quite tunable, there exists broad variability to control over dynamics of energy and charge carriers. 2,2’‐[[6,6,12,12‐Tetrakis(4‐hexylphenyl)‐6,12‐dihydrodithieno[2,3‐*d*:2’,3’‐*d’*]‐*s*‐indaceno [1,2‐*b*:5,6‐*b’*]dithiophene‐2,8‐diyl]bis[methylidyne(3‐oxo‐1*H*‐indene‐2,1(3*H*)‐diylidene)]]bis[propanedinitrile] (ITIC) was introduced to antisolvent to remove the excess ligands of perovskite QDs and form a heterointerfaces.^[^ ^139^ ^]^ Besides, ITIC covers the surface of perovskite QDs to stabilize the crystal phase. Benefitting from an effective charge transfer from perovskite QDs to ITIC, the *π*‐conjugated “charge driver” boosts the device PCE to 12.7%. Other conjugated molecules with suitable HOMO energy level can also fabricate a polymer‐QD heterojunction located at perovskite QD/HTL interfaces. Various conjugated ligands with dense *π*‐electrons such as PBDB‐T,^[^ ^140^ ^]^ non‐fullerene electron acceptor Y6‐F,^[^ ^141^ ^]^ 3‐phenyl‐2‐propen‐1‐amine (PPA), and DPPA^[^ ^98^, ^124^ ^]^ are introduced to perovskite QDs solution, which play an important role in improving the contact between the active layer and the HTL.

Generally, *π*‐electrons have significant contribution to the near‐edge conduction band when incorporated into low‐dimensional perovskite. However, to what degree do the conjugated ligands participate in the energetic ordering of states near the band extrema is still controversial. The lowered LUMOs are favorable to the charge separation in PSCs, but as for PeLEDs, the conjugated backbones should be designed precisely to avoid unnecessary nonradiative recombination.

### 3.3 Improving Phase Stability

**Figure 10:**
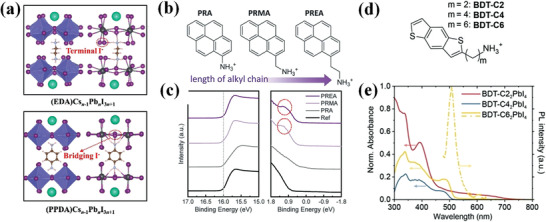
a) The enlarged patterns of A'Cs*_n_*_−1_PbnI_3_ *_n_*_+1_ (A' = EDA or PPDA) perovskites and the description of I—H bonds. Reproduced with permission.^[^ ^121^ ^]^ Copyright 2021, Wiley‐VCH. b) Chemical structures of three different organic ammoniums featuring different lengths of tethering alkylammonium. c) Ultraviolet photoemission spectroscopy results. Reproduced with permission.^[^ ^145^ ^]^ Copyright 2021, The American Association for the Advancement of Science. d) Benzodithiophene‐based ligand chemical structures. e) Corresponding UV/Vis absorption and PL spectra. Reproduced with permission.^[^ ^146^ ^]^ Copyright 2021, Wiley‐VCH.

Low‐dimensional perovskite with *π*‐conjugated ligands exhibits enhanced phase stability. There are multiple factors that can lead to improved stability with conjugated ligands. First, the hydrophobic, rigid, and bulky conjugated cations act as encapsulating layers to suppress ion migration, enhance moisture and environment stability while maintaining high conductivity compared to aliphatic cations. As a result, films with conjugated ligands are exceptionally stable both in water and air.^[^ ^126^, ^142^ ^]^ Besides, perovskite with conjugated molecule like PPDA, as mentioned above, exhibits extra phase stability compared with EDA.^[^ ^121^ ^]^ In PPDA‐intercalating perovskite, all of H in ‐NH_3_^+^ group bond with bridging I^−^ originating from the rigid phenyl ring, whereas in EDA‐intercalating perovskite two of H in ‐NH_3_^+^ group bond with terminal I^−^ (**Figure** **10**a). The bridging I^−^ is less likely to be displaced once exposed to ‐NH_3_^+^, leading to less structure distortion and higher phase stability. In addition, low‐dimensional perovskites with conjugated spacers are reported to stabilize the crystals via intermolecular interactions. Compared with PEA cations, Gao et al. observed the significantly improved stability of Sn‐based perovskite by incorporating conjugated 4Tm (Figure 9f).^[^ ^143^ ^]^ Although in layered perovskite, direct *π*–*π* stacking is not observed between 4Tm molecules, the conjugated 4Tm cations are probably responsible for shortened Sn—I bonds and shorter average N—I distance, which may provide extra driving force for the stabilization of the hybrid crystal. Park et al. reported Sn‐based 2D perovskite using stilbene derivatives 2‐(4‐(3‐fluoro)stilbenyl)ethanammonium iodide (FSAI) as the organic cations.^[^ ^142a^ ^]^ The FSA molecules in TI‐FSP with two phenyl rings are stacked by *π*–*π* interactions, along with additional supramolecular interactions such as hydrogen bonds, interlayer F···F interaction, and weak face‐to‐face type *π*–*π* interaction, all together make a compact layer. In contrast, the PEA organic cations with void spacers between them only have weak interchain interactions and the BA cations are similarly loosely packed in crystals.

Especially for PeLEDs, the conjugated molecules can promote the interaction between perovskite layers/dots to achieve better luminous properties, especially the device stability. Therefore, the lifetime of PeLEDs can be prolonged greatly. For example, by suppressing the presence of enormous surface bromide vacancies, the PL quantum yield of DBSA‐based perovskite QDs was maintained even after eight purification cycles and more than 5 months storage.^[^ ^101^ ^]^ Moreover, due to efficient carrier transfer with the assistance of *π*‐electrons, QDs/quasi‐2D perovskite devices with conjugated ligands such as ABABr (4‐(2‐aminoethy)benzoic acid),^[^ ^144^ ^]^ F‐pPEABr,^[^ ^125^ ^]^ P‐PDABr_2_ (1,4‐bis(aminomethy)benzene bromide),^[^ ^103^ ^]^ An‐HI (aniline hydroiodide),^[^ ^95^ ^]^ and PMA (poly(maleic anhydride‐alt‐1‐octadecene))^[^ ^115^ ^]^ exhibit higher current density and more stable luminous performance.

Although conjugated backbones are essential to improve the photoelectric properties of low‐dimensional perovskite, unconjugated alkyl tails also play a specific role in stabilizing the low‐dimensional structure. Xue et al. explored the bang‐edge states of conjugated organic cations featured different lengths of the tethering alkylammonium group—PRA, PRMA, and PREA (Figure 10b).^[^ ^145^ ^]^ Both PREA and PRMA could anchor into the organic layers and have additional electronic states near the tail of the VB, while PRA without flexible alkyl chain fails to insert into organic layers (Figure 10c). Besides, PRMA with a shorter alkyl chain shows a distorted intercalation configuration and disrupted *π*–*π* stacking. Guo and co‐workers found that through increasing the alkyl chain length, the stabilization effect of phenylalkylammonium iodide was enhanced because its binding with perovskite was stronger, and steric hindrance was increased for reconfiguration of accommodating ion migration.^[^ ^142b^ ^]^ The study carried out by Gao et al. suggests there exist other effects of alkyl groups on material properties.^[^ ^84^ ^]^ Compared with the short‐conjugated ligand containing a bithiophene unit (2T), the tetrathiophene ligand (4T) has a stronger intermolecular *π*–*π* interactions and poorer solubility. As a result, the author failed to obtain (4T)_2_PbI_4_ single crystal because of strong self‐aggregation. In order to solve this problem, two more sterically demanding methyl groups are incorporated to form a new ligand (4Tm) and successfully obtained (4Tm)_2_PbI_4_ single crystals. Darwich et al. in their recent works found the role of the linker length is critical for incorporation into 2D perovskite by preparing benzodithiophene (BDT)‐based cations with different alkane linkers (Figure 10d).^[^ ^146^ ^]^ The BDT‐C2‐based perovskite film shows an extended absorption shoulder until ≈700 nm, which is quite similar to the films of pure BDT and evidence of extending self‐aggregation (Figure 10e). The shoulder absorption is absent in BDT‐C4 and BDT‐C6 based films. Therefore, RP phases without *π*–*π* stacking are formed. As layered perovskite containing conjugated cations are limited by their *π*–*π* stacking, and the size of the cation is restricted by the perovskite octahedral, disrupting the self‐aggregation of the organic with steric groups needs to find a balance between out‐of‐plane conductivity and organic spacer incorporation.

To sum up, the intermolecular interaction between conjugated backbones and perovskite improves the perovskite phase stability. But the intermolecular *π*–*π* stacking between conjugated molecules is unfavorable to the formation of low‐dimensional perovskite. Therefore, alkyl tails with perfect lengths are needed to increase steric hindrance and insert deep into perovskite crystals. With the combination of conjugated backbones and alkyl tails, conjugated ligands interact closely with perovskite to fabricate stable low‐dimensional devices.

## 4 Other Applications

In addition to additives for 3D perovskite and ligands for low‐dimensional perovskite, other applications have been explored utilizing *π*‐conjugated molecules. Wang et al. blended the conjugated polymer poly[[4,8‐bis[(2‐ethylhexyl)oxy]benzo[1,2‐*b*:4,5‐*b*′] dithiophene‐2,6‐diyl][3‐fluoro‐2‐[(2‐ethylhexyl)carbonyl] thieno[3,4‐*b*]thiophenediyl]] (PTB7) with lead perovskite MAPbI_3_ via a two‐step method.^[^ ^147^ ^]^ The PbI_2_ and PTB7 thin films are previously deposited, followed by the spin‐coating of MAI solution. The PTB7 is favorable to efficient charge transfer and strong barrier protection within the blend. Compared with pure MAPbI_3_ devices, comparable PCE and enhanced stability are achieved for PTB7‐MAPbI_3_ solar cells. Yue et al. utilized a series of large conjugated organic cations as structural direction agents owing to its unique and diverse template effects.^[^ ^148^ ^]^ The large and rigid conjugated cations have more stable template effects than the saturated organic cations. Furthermore, the conjugated cations can facilitate electron delocalization and charge separation as well as broaden light absorption range. As a result, the prepared cuprous/lead with long range face to face *π*–*π* interactions has excellent visible light‐driven photocatalytic properties. Fillafer et al. selected ammonium‐modified azobenzene compounds and organic–inorganic hybrid perovskite as molecular switches.^[^ ^149^ ^]^ The combination of azobenzene molecules with semiconducting materials is promising in optical switching because of its chemical and physical changes associated with the switching process. The electronic system of a semiconductor can affect the symmetry‐allowed *π*→π* transition and the subsequent structural relaxation. Through efficient energy transfer between perovskite phase and azobenzene, perovskite can achieve novel photo switching performance by interacting with the azobenzene molecules.

## 5 Conclusion

Plenty of *π*‐conjugated additives have been applied to prepare halide perovskite devices with high efficiency and long‐term stability. As few systematic researches have been carried out on the mechanism of how *π*‐electrons affect perovskite active layers, we review multifarious *π*‐conjugated molecules adopted for halide perovskite additives and summarize their function as additives for 3D perovskite and ligands for low‐dimensional perovskite. Compared with alkyl‐based additives, *π*‐conjugated additives helped passivate perovskite defects, guide lattice orientation, rearrange energy level, and improve stability of perovskite in a superior way. On the other hand, conjugated ligands with superior electrical conductivity have promising prospects in low‐dimensional perovskite. It is expected that versatility of conjugated semiconductive additives and halide perovskites in photoactive properties will create various combinations with advantages offered by both of them.

It should be realized that there are few studies concerning the application of conjugated molecules on upscale perovskite films, which is a major research direction in the current development of PSCs. Considering the interaction mechanism with perovskite as we discussed in this article, it can be speculated that the introduction of conjugated additives can apply to upscale technique and help with upscale device performance. We also notice that only several reports are related to lead‐free perovskite, and most of them are about Sn‐based perovskite, which interacts with conjugated additives similar to Pb‐based perovskite. The interaction between conjugated additives with lead‐free perovskite mainly depends on the coordination ability of metal atoms. As Sn and Pb belong to the same group, their coordination abilities have many similarities. Elements in other groups which have empty p orbitals for coordination such as Sb, Bi might have similar coordination ability with conjugated molecules. But it is speculated that transition metals with empty d orbitals would interact differently. More research should be carried out to explore the interaction mechanism between conjugated molecules and lead‐free perovskite.

In the meanwhile, it is worth noticing that the self‐aggregation of conjugated molecules has significant influences on perovskite crystals, especially for low‐dimensional perovskite. If the self‐aggregation effect is not effectively suppressed, the conjugated molecules will stay close together rather than insert uniformly into the perovskite lattice. Besides, the introduction of conjugated ligands into PeLEDs will lead to decrease in the triplet exciton energy, which increases the possibility of nonradiative recombination. Therefore, the structure of conjugated ligands for low‐dimensional perovskite should be designed precisely.

In conclusion, tailoring functional conjugated molecules to perovskite has been proven as effective approaches to improve device performance and, more types of conjugated molecules should be explored as perovskite additives. In view of the role *π*‐electrons played in perovskite layers, we suppose that *π*‐conjugated additives with comparable size of perovskite octahedral can easily permeate into bulk perovskite and assemble with external perovskite crystals. Therefore, deeper researches focusing on this aspect deserve more attempts. Besides, considering conjugated ligands have noteworthy positive effects on the stability of 2D Sn‐based perovskite, stabilizing the Sn‐based perovskite phase with *π*‐conjugated molecules is of great significance to fabricate stable, lead‐free perovskite devices. What is more, further investigation about understanding the interaction between perovskite and conjugated molecules while altering the conjugated backbone can give a clear guidance to fully exert the superiority of *π*‐electrons.

## Conflict of Interest

The authors declare no conflict of interest.
